# A history of repeated antibiotic usage leads to microbiota-dependent mucus defects

**DOI:** 10.1080/19490976.2024.2377570

**Published:** 2024-07-21

**Authors:** Kertu Liis Krigul, Rachel H. Feeney, Supapit Wongkuna, Oliver Aasmets, Sandra M. Holmberg, Reidar Andreson, Fabiola Puértolas-Balint, Kateryna Pantiukh, Linda Sootak, Tõnis Org, Tanel Tenson, Elin Org, Bjoern O. Schroeder

**Affiliations:** a Estonian Genome Centre, Institute of Genomics, University of Tartu, Tartu, Estonia; b Department of Molecular Biology, Umeå University, Umeå, Sweden; c Laboratory for Molecular Infection Medicine Sweden (MIMS), Umeå University, Umeå, Sweden; d Umeå Centre for Microbial Research (UCMR), Umeå University, Umeå, Sweden; e Institute of Molecular and Cell Biology, University of Tartu, Tartu, Estonia; f Institute of Technology, University of Tartu, Tartu, Estonia

**Keywords:** Antibiotics, colonic mucosa, fecal microbiota transplantation, gut microbiome, intestinal barrier, mucus, short-chain fatty acids, Akkermansia

## Abstract

Recent evidence indicates that repeated antibiotic usage lowers microbial diversity and ultimately changes the gut microbiota community. However, the physiological effects of repeated – but not recent – antibiotic usage on microbiota-mediated mucosal barrier function are largely unknown. By selecting human individuals from the deeply phenotyped Estonian Microbiome Cohort (EstMB), we here utilized human-to-mouse fecal microbiota transplantation to explore long-term impacts of repeated antibiotic use on intestinal mucus function. While a healthy mucus layer protects the intestinal epithelium against infection and inflammation, using *ex vivo* mucus function analyses of viable colonic tissue explants, we show that microbiota from humans with a history of repeated antibiotic use causes reduced mucus growth rate and increased mucus penetrability compared to healthy controls in the transplanted mice. Moreover, shotgun metagenomic sequencing identified a significantly altered microbiota composition in the antibiotic-shaped microbial community, with known mucus-utilizing bacteria, including *Akkermansia muciniphila* and *Bacteroides fragilis*, dominating in the gut. The altered microbiota composition was further characterized by a distinct metabolite profile, which may be caused by differential mucus degradation capacity. Consequently, our proof-of-concept study suggests that long-term antibiotic use in humans can result in an altered microbial community that has reduced capacity to maintain proper mucus function in the gut.

## Introduction

The colon is densely populated by commensal bacteria that play an important role in aiding digestion and preventing colonization by pathogenic bacteria. However, if not controlled by the host, the intestinal bacteria can pose a threat. Therefore, a gel-like mucus layer, which lines the colonic epithelium, acts as a barrier to both the native bacteria and any potential pathogens by physically separating the epithelium from the luminal content. Moreover, while initially considered to be only a lubricant for fecal material, the mucus layer has since been shown to play an important role in host immune defense and immune cell signaling regulation, as well as acting as a reservoir for signaling peptides.^1^

A healthy colonic mucus layer can be described as having a gradient, consisting of a sterile and densely structured inner area, lying closest to the epithelium, and a more loosely structured outer area that is colonized by the native microbiota and periodically washed away along with fecal material, which itself can be covered by mucus derived from the proximal part of the colon.^2^ To thus replenish the layer, mucus is continuously secreted by goblet cells located in the epithelium, thereby creating a luminal-directed flow, referred to as the mucus growth rate, which is estimated as ~2 µm/min in healthy mice and ~4 µm/min in healthy humans.^3,4^ This mucus growth actively maintains a safe distance between the epithelium and the microbiota, and we recently showed that the microbially produced short-chain fatty acids (SCFAs) acetate and propionate directly stimulate mucus growth *ex vivo* from viable tissue through the activation of free fatty acid receptor 2 (Ffar2).^5^

The presence of the gut microbiota is known to be crucial for the development of a functioning mucus layer, as illustrated in germ-free mice where the lack of microbiota results in a dysfunctional, penetrable inner mucus layer.^6^ Moreover, the composition of the gut microbiota can affect mucus function and development.^7^ A change in composition can result in certain bacterial species beginning to degrade the mucus layer at an excessive rate, contributing to altered mucus structure which allows bacteria to move closer to the epithelium, consequently triggering inflammation.

Recent studies have shown that changes in gut microbiota composition, for example through consumption of a low-fiber diet, cause defects in the colon mucus layer^8,9^. Moreover, impaired mucus function has been observed in colitis, diabetes, and obesity in mice^10–13^ and has been connected to metabolic disease^14^ and ulcerative colitis^10,15^ in humans. Interestingly, the mucus defect may even precede the onset of ulcerative colitis and thereby contribute to its development.^15^

Besides diet, antibiotic intake has a major impact on gut microbiota composition.^16,17^ By assessing data collected from volunteers in the Estonian microbiome cohort (EstMB), we were able to recently show that a history of repeated antibiotic usage causes perturbations to the gut microbiota, including loss of diversity, which can persist over time.^18^ With the dramatic rise in global antibiotic use in recent years, an increase in various complex diseases, including asthma,^19^ allergies,^20^ celiac disease,^21^ diabetes,^22^ and chronic inflammatory diseases of the gastrointestinal tract,^23^ has been observed. Interestingly, maternal antibiotic-perturbed microbiota has been shown to exacerbate gut inflammation when transferred to mouse pups deficient in the anti-inflammatory cytokine interleukin 10 (IL10^−/−^),^24^ and antibiotic usage has been significantly associated with an increased risk of new-onset IBD,^25^ indicating a connection between antibiotic use, microbiome composition and gut health.

While our previous work revealed the significant impact of antibiotic usage on microbiome composition and links between microbiota changes and disease,^18^ little is known about the long-term impact of antibiotic treatment on mucosal barrier function. As antibiotics can modify microbiota composition and microbiota can, in turn, affect mucus characteristics, we hypothesized that antibiotic usage may lead to changes in the colonic mucus layer. To address this, we utilized our deeply phenotyped EstMB cohort and selected human microbiota samples from individuals with a history of repeated, but not recent, antibiotic usage and a matched control group. Using human-to-mouse fecal microbiota transplantation (FMT) alongside *ex vivo* mucus function assessment from viable tissue, we studied the impact of antibiotic-altered human microbiota on the mouse gut environment, with a particular focus on the colonic mucus layer.

## Materials and methods

### Human donor recruitment and sample collection

Human donors were recruited as part of the Estonian Microbiome (EstMB) project established in 2017, as described in detail previously.^18^ Briefly, 2509 volunteers from the Estonian Biobank, which contains more than 210,000 genotyped adults, donated stool, oral, and plasma samples for the microbiome study. The cohort currently includes 1764 female and 745 male participants, aged 23–89 years old. The participants collected a stool sample immediately after defecation and delivered it to the study center, where it was stored at −80°C until further processing. All participants provided informed consent and signed a broad consent form, which allows access to the participant’s personal and medical health record data from national health registries and databases. Additionally, patients reported their health-related behavior by completing a lifestyle questionnaire, which included questions about their physical activity, medical data, living environment, and stool characteristics (i.e. Bristol stool scale). Dietary habits were recorded through a food frequency questionnaire (FFQ) that included information about the consumption of coffee, tea, bread/white bread, potatoes, rice/pasta, porridge/muesli, dairy, fish, meat, meat products (wieners, sausages), fresh vegetables, boiled vegetables, fresh fruits, berries, compote/jams, sweets, soft drinks, eggs, and added salt. In addition, we assessed the participants’ general eating habits with the following options: Omnivore; Omnivore, excl milk and cream; Omnivore, excl fish; Omnivore, excl red meat; Vegan; Vegetarian eating dairy and eggs; Vegetarian eating fish; Vegetarian eating dairy products. The study was approved by the Research Ethics Committee of the University of Tartu (approval No. 266/T10) and by the Estonian Committee on Bioethics and Human Research (Estonian Ministry of Social Affairs; approval No. 1.1–12/17). The rights of gene donors are regulated by the Human Genes Research Act (HGRA) § 9 – Voluntary nature of gene donation.

### Human donor inclusion criteria

The following inclusion criteria were used for this study: Healthy adults of both sexes with normal BMI (between 18.5 and 25), no medical history of complex diseases (listed in **Supplementary Table S1**) and had not taken any medications in the 3 months prior to stool sample collection (listed in **Supplementary Table S2**) nor antidepressants in the 5 years prior to stool sample collection. Additionally, participants with seasonal allergies or celiac disease according to the questionnaire data were excluded.^17–26–27–28^ Likewise, vegans and vegetarians were excluded to avoid any diet-dependent bias on microbiota composition.

For the donor group of repeated antibiotics users (hABX), we identified four individuals that did not take any antibiotics in the 6 months prior to stool sample collection but had used antibiotics at least 5 times within the last 5 years. Of note, we excluded participants who had taken antibiotics for gastrointestinal diseases, to avoid any disease-related effects on the microbiome. Only four individuals remained after applying these strict selection criteria.

For the control group of human donors (hCTRL), only individuals who had not used antibiotics in the last 10 years were included. Out of these, we selected matched controls by considering the following factors: age, sex, BMI, Bristol stool scale (stool type), and dietary habits (matched case–control pairs based on the three first principal components of a comprehensive Food Frequency Questionnaire, explaining in total 37.65% of the variability between individuals). In summary, from 2509 individuals, four cases were selected and matched with four controls. For both treatment groups, stool samples from the respective four individuals were pooled and prepared for microbiota transplantation.

### Human-to-mouse fecal microbiota transplantation (FMT)

Male (*n* = 8) and female (*n* = 8) C57Bl/6J 10-week-old mice, originally obtained from Charles River Laboratory Germany, were bred in-house and kept in individually ventilated cages in a pathogen-free environment at 22 ± 1°C under a 12:12-hour light–dark cycle. All mice had *ad libitum* access to autoclaved water and food and were fed a standard chow diet (#801730, Special Diet Services, UK). For FMT, antibiotic pre-treatment to deplete inherent mouse bacteria was performed as previously described^29^ with modifications. Briefly, all mice were given autoclaved drinking water supplemented with an absorbable antibiotic cocktail (ampicillin (1 mg/ml), cefoperazone (0.5 mg/ml), clindamycin (1 mg/ml)) for 5 days, followed by a 2-day washout period. Next, a non-absorbable antibiotic cocktail (streptomycin (1 mg/ml), neomycin (1 mg/ml), vancomycin (0.5 mg/ml)) was administered for an additional 5 days, followed by a final 2-day washout period.

Inoculation with human microbiota using oral gavage was carried out directly after the second washout period. FMT gavage suspensions were prepared in an anaerobic chamber (Whitley DG250 Anaerobic Workstation) using 100 mg of human stool sample per 1 ml of PBS supplemented with 0.1% L-cysteine (Sigma-Aldrich, St-Louis, MO, USA) and 15% glycerol. The suspension was incubated for 10 min to allow large particles to settle. The four-case (hABX) and four-control (hCTRL) samples were pooled into two separate suspensions, and 200 µl of suspension per mouse was then gavaged into the corresponding group of antibiotic-pretreated mice (*n* = 4 males and *n* = 4 females per group, *n* = 16 in total). The gavages were repeated after 4 and 7 days. Three d after the final gavage (i.e. 10 days after the first gavage), the mice were sacrificed. This selected time point was a compromise to allow microbial establishment and development of a microbiota-dependent mucus phenotype but at the same time prevents outcompeting of the transplanted community by potential inherent mouse bacteria that had survived the antibiotic treatment.

After sacrifice, the distal colon was collected for immediate *ex vivo* analysis of mucus growth rate, mucus penetrability, and histology. Additionally, distal colon content was collected for metagenomic sequencing, cecal content was collected for metabolomics profiling, and mid-colon tissue was collected for RNA expression analyses. The distal colon content was obtained directly from the distal colon upon termination. In cases where the colon was empty (*n* = 2), stool samples that were collected 1 day before the sacrifice were used. Additionally, colon length, cecum weight, abdominal fat weight, and body weight were measured. The researchers were blinded to the case−control groups until the end of the analyses.

### Mucus thickness and growth rate measurements

Colonic mucus layer thickness and growth rate were measured as previously described.^4,^^9^ Briefly, the distal part of the colon was gently flushed with Kreb’s buffer (116 mM NaCl, 1.3 mM CaCl_2_, 3.6 mM KCl, 1.4 mM KH_2_PO_4_, 23 mM NaHCO_3_, and 1.2 mM MgSO_4_ (pH 7.4)) to remove luminal content and unattached mucus. The muscle layer was then removed, and colonic tissue was mounted in a horizontal perfusion chamber system supplemented with a continuous basolateral supply of RPMI 1640 Medium-Gibco (ThermoFisher Scientific). The surface was visualized by adding black 10 μm polystyrene microspheres (Polysciences, Warrington, USA) on top of the mucus. Kreb’s-mannitol (10 mM mannitol, 5.7 mM sodium pyruvate, and 5.1 mM sodium glutamate) was added apically for hydration. Mucus thickness was measured using a fine glass micropipette connected to a micrometer under a stereomicroscope (Olympus). The mucus growth rate (μm/min) was obtained by measuring mucus thickness at time points 0 and 45 min, and then calculating the change in mucus thickness per minute.

### Mucus penetrability measurements

Mucus penetrability was measured as previously described^4,9^ with modifications. Briefly, the distal colon tissue was prepared in the same way as described above for mucus growth measurements. After placing the tissue in the perfusion chamber, the epithelium was stained with Syto 9 (ThermoFisher Scientific, 1:500 in Kreb’s-mannitol buffer), and the mucus layer was stained with Wheat Germ Agglutinin (ThermoFisher Scientific, 1:20 in Kreb’s mannitol buffer) and Ulex Europaeus Agglutinin I (Vector Laboratories, 1:20 in Kreb’s-mannitol buffer) for 10 min in the dark, on ice. Subsequently, the tissue was washed with Kreb’s-mannitol buffer and 1 µm bacteria-sized fluorescent microspheres (ThermoFisher Scientific, 1:20 in Kreb’s-mannitol buffer) were added on top of the mucus. The tissue was incubated for a further 10 min in the dark, on ice, to allow time for the microspheres to settle onto the mucus layer. Excess microspheres were gently washed away with Kreb’s-mannitol buffer, and the tissue was then covered apically with fresh Kreb’s-mannitol buffer. The tissue was visualized by acquiring Z-stack images (5 µm steps) with a 20×/0.5 N-Achroplan water dipping objective on an upright Zeiss LSM 800 confocal microscope. Two to four Z-stack images per mouse were taken. The images were then exported and processed with Imaris (Version 9.9.0, Oxford Instruments) to map epithelium, mucus, and microspheres to isosurfaces. On average 916 ± 185 (min 501, max 1252) distances between individual microspheres and epithelial surfaces per image were extracted and mucus penetrability was quantified by analysis of microsphere distribution within the mucus layer. The average fraction of microspheres penetrating the areas within 10 µm and 50 µm distance to the colonic epithelium were plotted for each image from the mice.

### Tissue histology

For each mouse, a section of distal colon was fixed in Methacarn solution (60% methanol, 30% chloroform, and 10% glacial acetic acid) at room temperature for at least 1 week. Samples were then paraffin-embedded using Sakura Tissue Tek VIP (USA). For Alcian-Blue-Periodic acid-Schiff (AB-PAS) staining, 5 µm colon sections were prepared. In brief, the staining procedure included deparaffinization of the sections in xylene (VWR Chemicals) and rehydration in ethanol gradients (99%, 90%, and 70%) and water. Thereafter, the slides were placed in 3% acetic acid (VWR Chemicals) and then stained with Alcian-Blue (Sigma-Aldrich) for 20 min. Tissues were oxidized in 0.05% periodic acid (Sigma-Aldrich) before staining with Schiff’s reagent (Sigma-Aldrich) for 20 min. Mayer’s hematoxylin (Sigma-Aldrich) was used for nuclear visualization, and section dehydration was performed in water, ethanol steps (70%, 90%, and 99%), and xylene. The resulting stained tissues were mounted under coverslips using Pertex glue (Histolab), and images were captured with 20X magnification using Pannoramic Scan P250 Flash III BL/FL (3DHistech, Hungary). The number of goblet cells per crypt and crypt length was evaluated for at least 10 crypts per mouse by three blinded scientists. Moreover, mucus thickness from fixed tissue was measured at 10 locations per section by two blinded scientists.

### Colony-forming unit (CFU) counts

Stool samples were taken from mice at multiple time points for CFU counting: at baseline before the start of the experiment (Day 0), after the absorbable antibiotic treatment (Day 5), after the first washout period (Day 7), after the non-absorbable antibiotic treatment (Day 12), and after the second washout period (Day 14). Stool samples were weighed and mixed with 500 µl of sterile PBS. The samples were then plated on Brain Heart Infusion (BHI) agar plates and incubated under anaerobic conditions at 37°C for 2 days. Thereafter, the colonies were counted.

### RNA extraction and cDNA generation

A biopsy of the distal colon was collected after sacrifice, immediately snap-frozen in liquid nitrogen and stored at −80°C until extraction. For RNA extraction, the tissue was homogenized in a TissueLyser II (Qiagen, Germany) using stainless steel beads (5 mm; Qiagen). RNA was then extracted using a RNeasy Mini kit (Qiagen), following the manufacturer’s protocol. RNA concentration and quality were determined using a Nanodrop Lite Spectrophotometer (ThermoFisher Scientific). Per sample, 500 ng of RNA was reverse transcribed to cDNA with a High-Capacity cDNA Reverse Transcription Kit (ThermoFisher Scientific) and diluted 1:7 in nuclease-free water.

### Quantitative real-time PCR analysis

Mouse cDNA was amplified with gene-specific primers and HotStarTaq Master Mix Kit (Qiagen) (Table 1). The amplicons were cloned into a pGEM-T vector (Promega, WI) and transformed into competent DH5α *E. coli* cells. The plasmids were isolated using a Plasmid Mini Kit (Qiagen), *s*equenced using Sanger sequencing (Eurofins Genomics, Ebersberg, Germany), the target copy number/ng DNA quantified, and target-specific dilution series were prepared. The copy number of specific transcripts was determined by analyzing mouse cDNA in a 10 μl reaction mix consisting of 1× iQ SYBR® Green Supermix (Bio-Rad, USA), 0.2 μM of each primer, and 2 μl of template cDNA on a CFX Connect Real-Time System (Bio-Rad). Samples and plasmid standards were amplified using the following protocol: denaturation at 95°C for 3 min, followed by 35 cycles of denaturation at 95°C for 20 sec, gene-specific annealing temperature (see Table 1) for 40 sec and extension at 72°C for 1 min. A standard curve was generated for each gene of interest, and the transcript copy number in each sample was calculated using the Bio-Rad CFX Maestro software (v.2.3) and reported as copy number/10 ng RNA.

**Table 1.** Gene-specific primers and annealing temperatures used for quantitative real-time PCR analysis.

Gene name		Primer (5’-3’)	Annealing temperature (°C)
Muc2	F	GAACGGGGCCATGGTCAGCA	60
	R	CATAATTGGTCTGCATGCC	
Reg3γ	F	CCTCAGGACATCTTGTGTCTGTGCTC	68
	R	TCCACCTCTGTTGGGTTCATAGCC	
Dmbt1	F	GGGGATCTCCACTGTTATCTTTGA	60
	R	AGAATCTGTTCCATCTGTGGGA	
mLys	F	GGCTGGCTACTATGGAGTCAGCCTG	65
	R	GCATTCACAGCTCTTGGGGTTTTG	
mBD4	F	CCACTTGCAGCCTTTACCC	63.3
	R	GCCAATCTGTCGAAAAGCGG	
Lypd8	F	GCCTTCACTGTCCATCTATTT	60
	R	GTGACCATAGCAAGACATGCA	
ZO-1	F	CCACCTCTGTCCAGCTCTTC	55
	R	CACCGGAGTGATGGTTTTCT	
Claudin-1	F	TCCTTGCTGAATCTGAACA	53
	R	AGCCATCCACATCTTCTG	
TNF-α	F	ACGGCATGGATCTCAAAGAC	55
	R	AGATAGCAAATCGGCTGACG	
IL-1β	F	AACCTGCTGGTGTGTGACGTTC	55
	R	CAGCACGAGGCTTTTTTGTTGT	

### DNA extraction and metagenomic sequencing

DNA extraction and sequencing of the EstMB donor stool samples was carried out as described previously.^18^ For the mouse samples, as well as the human stool gavage samples, the microbial DNA extraction was performed using a DNeasy PowerSoil Pro Kit (Qiagen, Germany), according to the manufacturer’s instructions. The DNA concentration was measured using the Qubit® dsDNA Assay Kit in Qubit® 2.0 Fluorometer (Life Technologies, CA, USA). DNA quality control, library preparation, and shotgun metagenomic paired-end sequencing were performed by Novogene Bioinformatics Technology Co., Ltd. Samples were sequenced using the Illumina NovaSeq6000 platform alongside a single mock community sample and a negative control.

### Bioinformatics analysis and processing of the metagenomic sequencing data

Reads were trimmed for quality and adapter sequences using fastp^30^ with – length_required 5 and – cut_mean_quality 30. The host reads that aligned with the human (build GRCh38) and mouse (build GRCm39) genomes were removed using Bowtie 2^31^ with parameters – minins 200 and – maxins 400 and SAMtools.^32^ This resulted in a total of 763,608,660 paired reads from all mouse samples, excluding controls (*n* = 64, mean 11,931,385 ± 7,366,927 read length 2 × 150 bp) and 116,713,904 paired reads from human donor samples (*n* = 8, mean 14,589,238 ± 1,153,486, read length 2 × 150 bp). Additionally, 14,404,157 reads were obtained from the hCTRL pool sample, and 26,510,699 reads were obtained from the hABX pool sample. As expected, the read numbers after the second washout (*n* = 16, mean 219,232 ± 254,232) were exceptionally low compared to the rest of the samples (*n* = 61, mean 15,884,751 ± 3,294,077), leading us to exclude these data from further analysis. We did not rarefy the counts to avoid loss of data. The taxonomic composition of the decontaminated and quality-filtered metagenomes was identified by using MetaPhlAn 4^33^ with default parameters and database version mpa_vOct22_CHOCOPhlAnSGB_202212. In total, 20 phyla, 858 genera, and 1277 species were identified from the sample set.

Additionally, host-cleaned reads from human donors and distal colon content samples were also used for *de novo* metagenomic assembly to detect *Akkermansia muciniphila* strains from these samples. First, the reads were assembled into contigs with MEGAHIT v1.2.9.^34^ Thereafter, the contigs were binned using single binners – MetaBAT v2.15,^35^ Maxbin v2.2.7^36^ and VAMB v3.0.7.^37^ Since different binning tools reconstruct genomes at various levels of completeness, bin aggregation software, i.e. DAS Tool v1.1.4,^38^ was used to integrate the results of bin predictions made by VAMB, MetaBAT2, and MaxBin2 to optimize the selection of non-redundant, high-quality bin sets using default parameters. Bin quality, including completeness and contamination, was estimated using CheckM v2.^39^ Bin statistics, including total size, number of contigs, N50, and GC content, were obtained using seqkit v2.3.1.^40^ Finally, bins were taxonomically annotated using GTDB-Tk v2.3.0, GTDB release number 214.^41^ The taxonomic position of the assembled genome was determined using GTDB-Tk. All assembled genomes belonging to the *Akkermansia muciniphila* species were clustered at strain level with an average nucleotide identity (ANI) threshold of 99%, as calculated with FastANI v2.09.^42^ The clustering procedure resulted in two strain-level clusters. The best quality genome from each strain cluster was selected as its representative based on genome completeness, minimal contamination, strain heterogeneity, and assembly N50. The reads from distal colon content and human samples were then mapped against the representative genomes from each cluster.

### Metabolomics analysis

Metabolomics profiling was carried out at the FIMM Metabolomics Unit at the University of Helsinki, Finland, and the scientists were blinded to the sample groups. Metabolites were extracted from 30 mg of cecum samples with 400 µL of cold extraction solvent (Acetonitrile: Methanol: Milli-Q Water; 40:40:20, ThermoFisher Scientific). Subsequently, the samples were homogenized in three cycles of 30 sec at 5000 rpm at 4°C, centrifuged and supernatants were passed through a Phenomenex Phree Phospholipid removal 96 well plate using robotic vacuum. Sample filtrates were transferred into evaporation tubes and dried under a gentle stream of nitrogen. The dried samples were then re-suspended with 50 µL of cold extraction solvent (Acetonitrile: Methanol: Milli-Q Water; 40:40:20) and analyzed for targeted relative profiling and for relative SCFA abundance on a Thermo Vanquish UHPLC coupled with a Q-Exactive Orbitrap quadrupole mass spectrometer, equipped with a heated electrospray ionization (H-ESI) source probe (ThermoFisher Scientific). Scanning was performed using the full MS and polarity switching modes in the mass range 55–825 m∕z and the following settings: resolution of 70,000, spray voltages: 4250 V for positive and 3250 V for negative mode, sheath gas: 25 arbitrary units (AU), and auxiliary gas: 15 AU, sweep gas flow 0, Capillary temperature: 275°C, S-lens RF level: 50.0. Instrument control was operated with Xcalibur 4.1.31.9 software (ThermoFisher Scientific).

The chromatographic separation for metabolite profiling was performed using a SeQuant ZIC-pHILIC (2.1 × 100 mm, 5-μm particle) column (Merck) at 40°C, with a flow rate of 100 µl/min and a total run time of 24 min. Gradient was started with 2 min at 80% mobile phase B (Acetonitrile) and then gradually increased to 80% mobile phase A (20 mM ammonium hydrogen carbonate in water, adjusted to pH 9.4) until 17 min, back to 20% A at 17.1 min and then equilibrated to the initial conditions for 7 min.

For SCFA profiling, the separation was performed using a Hypercarb Porous Graphitic Carbon HPLC Column, 50 × 2.1 3 μm (ThermoFisher Scientific) with the gradient starting at 2.5 min at 100% mobile phase A, gradually increased to 100% mobile phase B for 10 min, held until 18 min, returned to 100% A at 18.1 min, and then equilibrated to the initial conditions for 6 min. TraceFinder 4.1 software (ThermoFisher Scientific) was used for data integration, the final peak integration and peak area calculation of each metabolite. Data quality was monitored throughout the run using pooled samples such as Quality Control (QC), prepared by pooling each study sample, which was interspersed after every 10th sample throughout the run.

Out of the targeted metabolomics panel with 462 metabolites, 220 metabolites were detected in our samples. The metabolite data were checked for peak quality (poor chromatograph), RSD (%relative standard deviation, 20% cutoff), and carryover (20% cutoff). Additionally, metabolites with multiple missing values were excluded from the analysis. After quality control, 171 metabolites remained for further analysis. No samples were excluded from the data analysis. The identified metabolites were normalized according to sample weight before analyzing the data (peak intensities) using Metaboanalyst 6.0 (www.metaboanalyst.ca.).

The metabolomics profile data were transformed using log-transformation. Data scaling was carried out for each variable by mean-centering the variable divided by the square root of the standard deviation (i.e. auto-scaling). Significantly different metabolites between the two groups were identified using the Wilcoxon Rank Sum test. Metabolites with a fold change of 1.5 and an FDR < 0.05 were considered significant.

The same quality procedures were applied to the SCFA metabolomics panel, and all four metabolites passed quality control. The metabolites were also normalized according to sample weight before analyzing peak intensities, and significantly different metabolites between the two groups were identified using the Wilcoxon Rank Sum test.

### Statistical analyses

If not stated otherwise, the data analysis was completed using GraphPad Prism 8. For comparisons between the groups, an unpaired t-test was used when samples were distributed normally, as indicated by the Shapiro–Wilk test, and the Mann−Whitney U/Wilcoxon Rank Sum Test was used for non-normally distributed samples. In the case of assessing sex differences in body characteristics between the FMT-ABX and FMT-C groups, comparisons were carried out with multiple t-tests. For correlation analyses, Pearson correlation coefficients were computed for normally distributed data, as determined by the D’Agostino & Pearson test, while Spearman correlation coefficients were computed for non-normally distributed data. P-values were corrected for multiple comparisons where appropriate, according to the Benjamini–Hochberg procedure (False Discovery Rate (FDR) < 0.05). In all figures, data are presented as mean ± SD.

Additional analyses were performed using different packages in the R environment (version 4.3.1), as described in further detail below. In all R analyses, stringr (version 1.5.0) and tidyverse (version 2.0) packages were used for data manipulations and ggplot2 (version 3.3.6), ggsci (version 2.9), and ggpubr (version 0.4.0) packages were used for data visualizations.

To evaluate whether there are significant differences in mucus penetrability between the two groups, the linear mixed-effects model was used by applying the *lmer* function in the lmerTest package (version 3.1.3) on bead-distance measurement values, with the study group as a fixed parameter and mouse and image number as nested random parameters. This allowed us to account for the dependencies in data arising from different numbers of observations per mouse and image.

For the microbiome analyses, the R package phyloseq (version 1.46.0) was used to import, store, and analyze the data. The observed number of unique species (richness) and the Shannon diversity index were used to assess the alpha diversity using the vegan package (v2.6.4). The Euclidean distance on the centered log ratio (CLR)-transformed microbiome species profile was used to calculate the between-sample distances for the beta diversity analysis. Permutational analysis of variance (PERMANOVA) on Euclidean distances was carried out using the *adonis* function in the vegan package to test the associations between the groups and microbiome composition using 10,000 permutations for the *p*-value calculations. Alpha and beta diversity analyses were performed on the whole identified composition. To test the differential abundance of different species, analysis of Compositions of Microbiomes with Bias Correction (ANCOM-BC, package version 2.4.0) and ANOVA-Like Differential Expression (ALDEx, package ALDEx2 version 1.34.0) were used. To limit the number of tests carried out when studying differentially abundant species, the species detected in at least two samples, per sample type, with mean relative abundances higher than 0.1% were selected, resulting in 105 species being tested in distal colon content samples. To account for multiple tests the Benjamini – Hochberg procedure was applied (FDR < 0.05).

## Results

**Figure 1.**
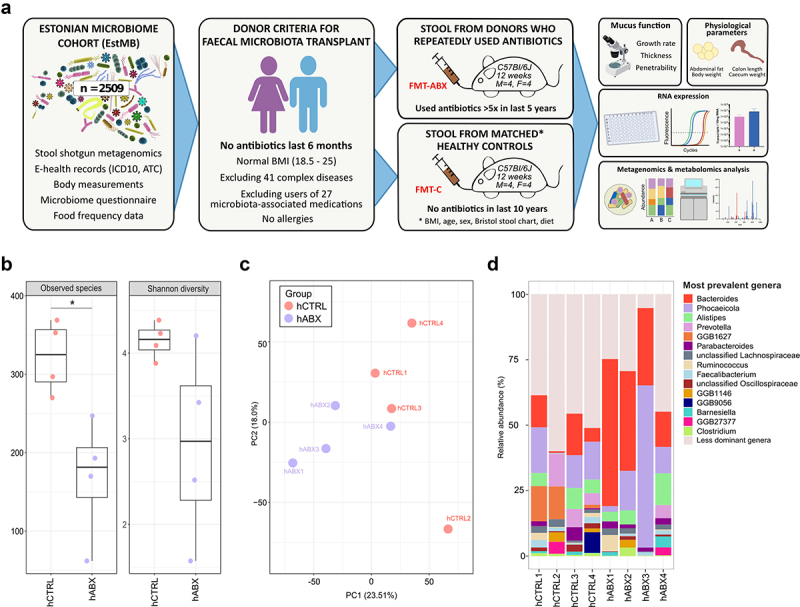
Overview of the donor selection, experimental plan and donor microbiomes. (a) Workflow of fecal microbiota donor selection from the EstMB participants, including mouse sample collection and analysis; (b) Alpha diversity analyses (observed number of species and Shannon diversity index) of individual human donor stools (hCTRL, hABX); (c) Beta diversity of the individual human donor stools and pooled donor stool; (d) Relative abundance of most common genera in hCTRL and hABX individual donors; hCTRL – human controls with no history of antibiotic use in 10 years preceding stool collection; hABX – human donors with a history of repeated antibiotic use. P-value corresponds to unpaired t-test, *p* < 0.05 (*).

To investigate the potential effect of gut microbiota changes caused by long-term antibiotic use,^18^ we carefully selected human subjects from the EstMB cohort using extensive Electronic Health Records (EHR), a Food Frequency Questionnaire, self-reported microbiome questionnaire data, and stringent exclusion criteria (Figure 1(a), **Supplementary Tables S1 & S2)**. Briefly, from 2509 Estonian Microbiome Project participants, four subjects who had not used antibiotics in the 6 months prior to stool sample collection but did use at least five courses of antibiotics in the last 5 y (hABX), and four subjects with no antibiotic usage history during the last 10 y (hCTRL) were selected. The hCTRL donors were matched with hABX donors based on BMI, sex, age, stool type (Bristol stool scale), and diet, as these factors are known to be major drivers of microbiome variability (Table 2). All selected participants were healthy, with normal BMI and no history of complex diseases (41 excluded diseases, **Supplementary Table S1**) nor any history of recent medication usage (27 excluded medications, **Supplementary Table S2**) that had been previously associated with the microbiome composition.^18^

**Table 2.** Characteristics of the FMT donors.

FMT donor Group	Antibiotics users				Controls				
Patient ID	hABX1	hABX2	hABX3	hABX4	hCTRL1	hCTRL2	hCTRL3	hCTRL4	p
Gender	Male	Female	Female	Female	Male	Female	Female	Female	1^i^
Age at sampling	30	51	30	52	39	51	31	59	0.56^ii^
BMI	21.06	24.69	23.14	24.94	24.49	18.83	23.03	23.04	0.34^ii^
Stool type (BSS)	1	3	3	4	1	4	2	5	NA
Antibiotics courses (last 5 y before sampling)*	5	7	10	10	0	0	0	0	0.02^i^
Diet	Omnivore	Omnivore	Omnivore	Omnivore	Omnivore	Omnivore	Omnivore	Omnivore	p > 0.05^i^

*- Controls had not taken antibiotics in the last 10 years. See the list of preceding exclusion criteria for donor selection in Methods and Supplementary Tables S1 and S2. BMI – body mass index, BSS – Bristol stool scale.

^i^- p-values based on Fisher’s exact test. ^ii^ - p-values based on the Wilcoxon Rank Sum Test. Diet was compared using the top three Principal Components which described the most variability in the Food Frequency Questionnaire.

Interestingly, although both donor groups were generally healthy and did not use antibiotics for at least 6 months prior to stool sample collection, analysis of microbial communities identified a significantly lower number of observed microbial species (168.0 ± 77.69 [hABX] versus 322.2 ± 46.54 [hCTRL]; *p* = 0.02) (Figure 1(b)) and significantly reduced Chao1 diversity (*p* = 0.02, **Supplementary Figure S1**) in the hABX donors compared to the hCTRL donors. A similar, yet not statistically significant, trend was observed for the Shannon diversity (2.9 ± 1.13 [hABX] versus 4.1 ± 0.21 [hCTRL]; *p* = 0.11) (Figure 1(b)) and the Simpson diversity index (*p* = 0.15, **Supplementary Figure S1**). Furthermore, the principal component analysis biplot shows the clustering of these two groups though this difference did not reach statistical significance according to the permutational analysis of variance (PERMANOVA R^2^ = 0.1856, *p* = 0.085) (Figure 1(c)). Analysis of gut microbial composition at genus level revealed several significantly different genera between hABX and hCTRL samples, including *Bacteroides*, *Phocaeicola, Parabacteroides, Blautia, Clostridium*, and *Akkermansia* among others (*p* < 0.05, Figure 1(d), **Supplementary Table S3**). Importantly, we observed that the donors chosen for this study exhibit similar changes in *Bacteroides* abundance (mean relative abundances 35.27 ± 18% in hABX donors versus 9.24 ± 7.3% hCTRLs) to those observed across the entire EstMB population cohort, where antibiotic usage resulted in more *Bacteroides*-dominant communities.^18^

### Repeated antibiotic use in humans promotes mucosal barrier dysfunction in mice following FMT

**Figure 2.**
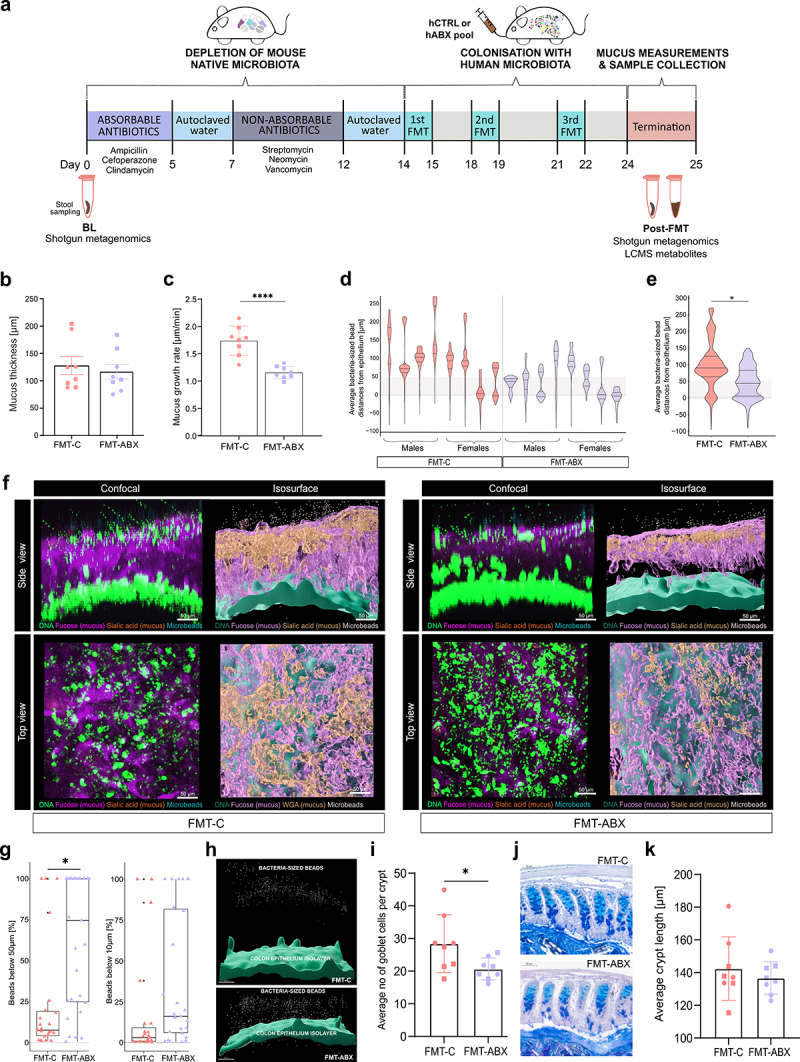
Mucus function analyses of mouse distal colon following FMT. (a) Outline of the mouse experiment; (b) Mucus thickness (µm) after gentle flushing of loose material; (c) Mucus growth rate (µm/min, p-value corresponds to unpaired t-test); (d) Average bacteria-sized bead distances from epithelium per mouse (µm); (e) Average bacteria-sized bead distances from epithelium per group (µm, p-value corresponds to linear mixed effects model); (f) Representative confocal Z-stack and isosurface images of the mucosal layer and epithelium in each group; (g) Percentage of beads within 50 µm and 10 µm from the epithelium in each measured image (2–4 images per mouse), per group (p-value corresponds to linear mixed effects model); (h) Representative isosurface images visualizing the bead distances from the epithelial isosurface; (i) Average number of goblet cells per crypt (p-value corresponds to Wilcoxon Rank Sum Test); (j) Representative histological images of AB-PAS stained colon tissue to visualize colonic crypts and goblet cells; (k) Average colonic crypt lengths. FMT – Fecal Microbiota Transplant, hCTRL pool – pooled stool from human controls with no history of antibiotic use in 10 years preceding stool collection, hABX pool – pooled stool from human donors with a history of repeated antibiotic use, BL – mouse baseline stool, FMT-C – mice that received FMT from hCTRL pool, FMT-ABX – mice that received FMT from hABX pool. *p* < 0.05 (*); *p* < 0.01 (**); *p* < 0.001 (***); *p* < 0.0001(****). Circles represent male mice while squares represent female mice.

To investigate whether long-term antibiotic-shaped gut microbiota could contribute to intestinal mucus dysfunction, stool samples from the hABX and hCTRL participants were pooled in two separate suspensions, according to the respective groups, in preparation for fecal microbiota transplantation (FMT). Each FMT group consisted of eight mice (four males and four females), and all mice received the same standard chow diet before and throughout the experiment. Groups of mice receiving microbiota from hABX and hCTRL donors were named FMT-ABX and FMT-C mice, respectively (Figure 1(a)). FMT was performed on microbiota-depleted young adult mice as, unlike germ-free mice, they have undergone normal development of the mucus layer and do not have an under-stimulated intestinal immune system.^43,44^ Following three repeats of the FMT, colonic mucus function was analyzed *ex vivo* on viable tissue 10 d after the first human-to-mouse transplant (Figure 2(a)).

Mucus growth is an intrinsic part of mucus function, which helps to maintain the mucus layer by providing a constant flow from the epithelial surface toward the lumen, thereby pushing microbes and debris away from the proximity of the epithelium. Thus, to compare the mucus growth between the two microbiota-transplanted groups, we measured the mucus thickness of the inner mucus layer periodically over 45 min to calculate the mucus growth rate. While the mucus thickness did not differ between the two groups after gently flushing away loose luminal material (116 µm ± 38 [FMT-ABX] versus 128 µm ± 47 [FMT-C]; *p* = 0.5824) Figure 2(b)), the mucus growth rate was significantly lower in the distal colon of hABX mice that received FMT from donors with antibiotic use history (hABX), compared to FMT-C mice that received FMT from healthy controls (hCTRL) (1.16 µm/min ± 0.11 [FMT-ABX] versus 1.74 µm/min ± 0.27 [FMT-C]; *p* < 0.0001) (Figure 2(c)), allowing the possibility for the microbes to move closer to the epithelium as the mucus flow is not pushing the microbes away as effectively.

While a healthy colonic mucus layer is impenetrable to bacteria, microbes can gain access to the colonic epithelium through degradation and penetration of this glycan-rich gel matrix. To assess mucus penetrability, we collected confocal Z-stack images of mouse distal colon tissue to determine mucus structure and location of bacteria-sized microspheres (i.e. beads) in the mucus. When measuring the distances of individual beads to the colonic epithelium surface, most of the beads were situated closer to the epithelium in FMT-ABX mice compared to the FMT-C group (Figure 2(d)). Further, the average distance of the beads from the epithelium was significantly lower (*p* = 0.025) in the FMT-ABX mice (47 ± 47 µm; median 45 µm) when compared to the FMT-C mice (98 ± 68 µm; median 90 µm) (Figure 2(e)). Likewise, physical changes to the mucus layer were observed in the top and the side view of the confocal microscopy Z-stack images, with a sparser mucus layer in the FMT-ABX group compared to the FMT-C group (Figure 2(f)).

In healthy mice, the inner ~50 µm of the mucus is generally considered impenetrable to microbes.^45^ However, we observed that an average of 61 ± 39% beads (median 74.55%) were closer than 50 µm from the epithelial surface in the FMT-ABX group, while in the FMT-C group only an average of 23 ± 34% (median 7.51%) were found to be in this vicinity (*p* = 0.038, Figure 2(g)). These findings indicate structural damage to the mucus layer of mice colonized by microbiota from humans repeatedly exposed to antibiotics. A similar tendency was observed for the area within 10 µm from the epithelial surface (average 35 ± 40% beads (median 16%) in the FMT-ABX group vs average 17 ± 32% (median 3%), in the FMT-C group), though this difference in proportions of beads did not reach statistical significance (*p* = 0.22, Figure 2(g)). Correspondingly, visualizing the epithelial isosurface and the beads illustrated that the beads were indeed more spread throughout the mucus layer in the FMT-ABX group compared to the FMT-C group, where beads were instead located at a greater distance from the epithelial isosurface (Figure 2(h)).

Furthermore, the counting of mucus-producing goblet cells indicated that the FMT-ABX group had a lower number of filled goblet cells compared to the FMT-C group (*p* = 0.035, Figure 2(i)). Yet, the analysis of histological sections did not reveal any signs of inflammation in either group (Figure 2(j)), which was also supported by similar lengths of the colonic crypts (*p* = 0.57, Figure 2(k)). Moreover, measuring mucus thickness from the fixed tissue sections confirmed a similar thickness between the two transplanted mice groups (*p* = 0.195, **Supplementary Figure S2F**) yet revealed that tissue fixation led to a reduction of the actual mucus thickness by around 90%. Overall, these results indicate a change in the physical structure of the mucus layer in the FMT-ABX group, allowing microbes to move closer to the epithelial layer compared to the FMT-C group.

### Increased expression of Muc2 and Reg3γ might compensate for impaired mucus function

**Figure 3.**
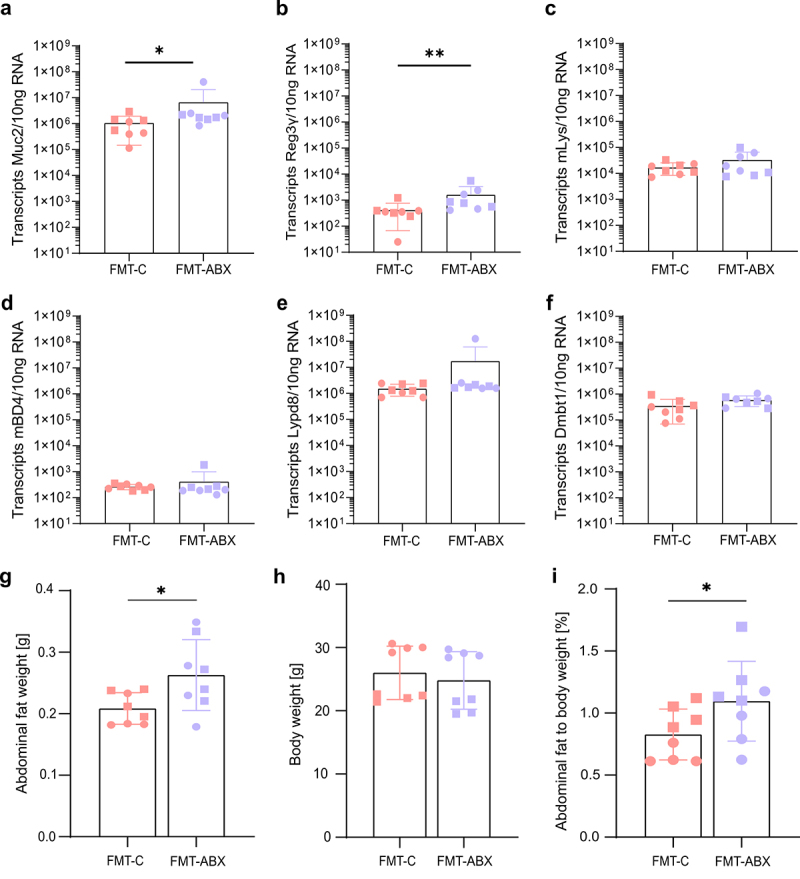
Expression of mucosal barrier function genes and physiological parameters following FMT. Absolute quantification of mRNA expression levels of (a) Mucin 2 (Muc2); (b) Regenerating islet-derived protein 3-gamma (Reg3γ); (c) Lysozyme (Lys); (d) Mouse Beta-defensin 4 (mBD4); (e) LY6/PLAUR domain containing 8 (Lypd8); (f) Deleted in malignant brain tumors 1 (Dmbt1) in mouse mid-colon tissue; (g) Abdominal fat weight (unpaired t-test) and (h) total body weight after FMT; (i) Abdominal fat as a percentage of total body weight (Wilcoxon Rank Sum Test); FMT – Fecal Microbiota Transplant. All p-values correspond to the Wilcoxon Rank Sum Test. *p* < 0.05 (*); *p* < 0.01 (**). Circles represent male mice while squares represent female mice.

As mucus function was impaired in the FMT-ABX mice compared to the FMT-C mice, we thus wondered whether the deterioration in host defense led to compensatory activation of alternative intestinal defense mechanisms to prevent the bacteria from moving closer to the epithelium. The host can respond to bacterial proximity to the colonic epithelium by increasing the secretion of antimicrobial peptides (AMPs), including defensins, lysozyme, regenerating islet-derived protein 3 gamma (Reg3γ) and Ly6/PLAUR domain containing 8 (Lypd8).^46–48^ As such, we assessed the expression of Mucin 2 (Muc2) and these AMPs using absolute quantification of RNA from colonic tissue samples. Expression of Muc2, the main component of mucus, was significantly higher in the FMT-ABX group compared to the FMT-C group (*p* = 0.02, Figure 3(a)). In addition, the expression level of the antibacterial lectin Reg3γ was significantly higher in the FMT-ABX group (mean 1.6 × 10^3^ transcripts/10 ng RNA [FMT-ABX] versus 4.1 × 10^2^ transcripts/10 ng RNA [FMT-C]; *p* = 0.003, Figure 3(b)). However, the expression of additional intestinal mucosal host defense proteins, such as lysozyme (Lys), mouse beta-defensin 4 (mBD4), Lypd8, or Deleted in malignant brain tumors 1 (Dmbt1) was not significantly different between the two groups (Figure 3(c)–(f)). Likewise, the expression of the tight junction proteins Zonulin-1 (ZO-1) and Claudin-1, as well as inflammatory markers TNF-α and IL-1β was also not significantly different between the two groups **(Supplementary Figure S3)**. Consequently, the microbiota-mediated mucus defect led to a selective compensatory host response toward encroaching gut bacteria. Moreover, linking the expression data of Muc2 with our mucus function measurements demonstrates that measuring Muc2 expression without any functional mucus analysis can be misleading, as it disregards the relevant post-transcriptional processing of mucus proteins that influence mucus properties and function.

Antibiotic-mediated changes in the gut microbiota are linked to metabolic impairments in humans and mice,^23^ and a defective mucus layer has been linked to a microbiota-dependent increase in abdominal fat in mice.^49^ To thus characterize whether the transplanted microbiome shaped by a history of antibiotic usage affected the mouse metabolism, we assessed the weight of abdominal fat and total body weight. Strikingly, we observed a significantly higher weight of abdominal fat in FMT-ABX mice (*p* = 0.028, Figure 3(g)), despite total body weight not differing between the two groups (Figure 3(h)). Furthermore, the percentage of abdominal fat contributing to the total body weight was significantly higher in FMT-ABX mice (*p* = 0.037, Figure 3(i)). These findings thus further corroborate a link between gut microbiota, defective intestinal mucosal barrier function, and metabolic impairments in the host.

### Distal colon microbiome composition differs between antibiotic users and healthy controls

**Figure 4.**
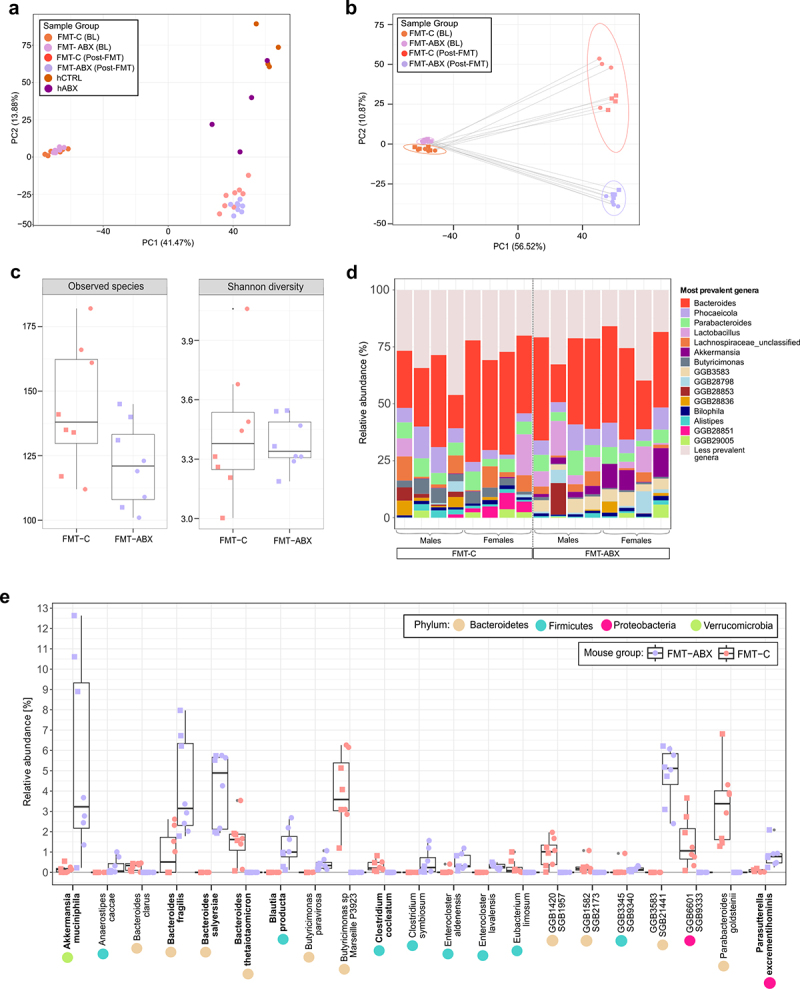
Distal colon microbiome composition of mice following FMT. (a) Beta diversity of the microbiota between FMT-C and FMT-ABX mouse stool at baseline, post FMT, and human donor stool used for FMT; (b) Beta diversity of the microbiota between FMT-C and FMT-ABX mouse stool at baseline and post FMT; (c) Boxplots representing alpha diversity (observed species and Shannon diversity index) of the mouse microbiota at termination; (d) Most prevalent genera in FMT-C and FMT-ABX gut microbiota of individual mouse samples; (e) Relative abundances of significant differentially abundant species in mouse microbiota between the two groups (FDR <0.05). Bacterial species previously shown to degrade mucus are indicated in bold. FMT – Fecal Microbiota Transplant. BL – mouse baseline stool, hCTRL – human controls with no antibiotic use history in last 10 y, hABX – human donors with a history of repeated antibiotic use, FMT-ABX – mice receiving FMT from hABX pool, FMT-C – mice receiving FMT from hCTRL pool. *p* < 0.05 (*). Circles represent male mice while squares represent female mice.

To next identify the differences in the microbial communities between the FMT-ABX and FMT-C groups, we initially evaluated the success of the microbiota transplant. Depletion of the inherent mouse microbiota prior to FMT by antibiotic cocktails was confirmed by stool plating, displaying a reduction in colony-forming units (CFU/g of stool) by at least 6 logs (**Supplementary Figure S4A**). Additionally, through plating of the pooled samples/FMT material, we confirmed that the microbes in the pooled donor samples were viable in both groups (>10^8^ CFU/g of pooled stool). Shotgun metagenomic sequencing revealed that 74 of the 113 species, around 65%, detected in human donors, were transferred to mice **(Supplementary Figure S4B**), which is in agreement with previous studies using a similar approach.^29,50^ Moreover, the sequencing analysis further identified that the inherent mouse microbiota before depletion (baseline, BL) was significantly different from human donor samples (PERMANOVA R^2^ = 0.5319, *p* < 0.00005), as well as from the mice microbiota 10 d post-FMT with human microbiota (PERMANOVA R^2^ = 0.56335, *p* < 0.00005). Correspondingly, the baseline mouse microbiota is separated from the human donors and mouse termination samples as shown by the first principal component (Figure 4(a)), indicating that the mouse microbiota becomes more similar to the human microbiota, which was also confirmed by Aitchison’s distance analysis (**Supplementary Figure S4C**). Crucially, the Euclidean distance metric-based beta diversity analysis of the CLR-transformed species-level profile confirmed that the FMT transplant led to a significantly different and donor-group specific microbiome composition of the mice (Figure 4(b), PERMANOVA R^2^ = 0.3163, *p* < 0.0002). Similar to the differences seen in the human donor groups, a lower number of species was observed in the FMT-ABX mice compared to the FMT-C mice though the difference was not statistically significant (*p* = 0.056), as was also the case for the Shannon (Figure 4c, *p* = 0.68), Chao1 (**Supplementary Figure S4D**, *p* = 0.056) and Simpson diversity indices (**Supplementary Figure S4E**, *p* = 0.26).

While no striking overall differences between individual FMT-ABX and FMT-C microbiota were observed for the most common genera (Figure 4(d)), compositional profiling at the species level revealed that 21 species from 4 different phyla were differentially abundant between the two groups (FDR < 0.05, Figure 4(e)). Of these 21 species, 12 were more abundant in the FMT-ABX group and 9 in the FMT-C group. In fact, the nine species were found to be unique to the FMT-C group, having not been detected in the FMT-ABX samples, which may indicate that these species play a protective role against the development of a mucus-deteriorating phenotype (Figure 4(e)). Conversely, out of the 12 species that were enriched in the FMT-ABX group, 6 could not be detected in any of the FMT-C samples (Figure 4(e)), again indicating that antibiotic usage might induce compositional changes, resulting in communities distinct from those of healthy controls. Interestingly, multiple species that were dominant in the FMT-ABX group have been previously shown to be mucin utilizers, including *Akkermansia muciniphila*, *Blautia producta*, *Parasutterella excrementihominis*, and species of the *Bacteroides* genus, such as *Bacteroides fragilis* and *Bacteroides salyersiae*.^51,52^ Previously reported mucin-utilizer species, i.e. *Bacteroides thetaiotaomicron*^53^ and *Clostridium cocleatum*^54^ were also found to be significantly more abundant in the FMT-C group; however, their relative abundance in the community was much lower compared with the mucin utilizers in the FMT-ABX group, which seemed to dominate in the community.

### History of antibiotic use results in a distinct microbial metabolite profile

**Figure 5.**
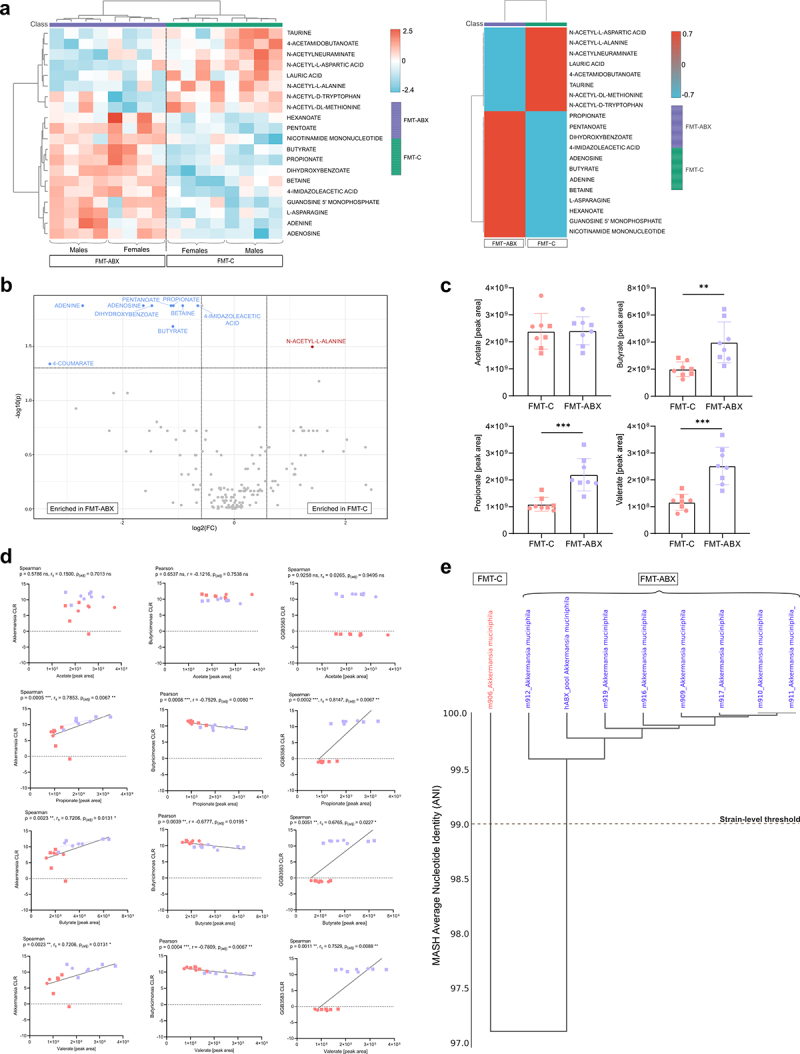
Metabolomic profiling of mice following FMT. (a) Individual mouse (left) and FMT-grouped (right) heatmaps of the top 20 metabolites from the relative metabolomics profile (unpaired t-test *p* < 0.05); (b) Volcano plot highlighting significant differentially abundant metabolites in the relative metabolomics profile (FDR <0.05); (c) Targeted SCFA metabolomics analysis of acetic acid, propionic acid, butyric acid and valeric acid; (d) Correlation between SCFA peak area and CLR of *Akkermansia, Butryicimonas* and *GGB3583,* p-values correspond to Spearman (r_S_) or Pearson correlation (r), as noted, p-value correction was performed using the Benjamini-Hochberg procedure (FDR <0.05). All other p-values correspond to the Wilcoxon Rank Sum Test. CLR = Centred Log Ratio; FC = fold change. *p* < 0.05 (*); *p* < 0.01 (**); *p* < 0.001 (***). Circles represent male mice while squares represent female mice.

The health status of the gut environment is determined by both the gut microbiota and the host immune system, as well as the interactions between them. The gut microbiota signal to the host via the production of metabolites.^55^ To thus analyze whether the history of antibiotic usage affects the microbial metabolomics profile in the transplanted mice, we carried out relative metabolomics profiling on mouse cecal content using high-performance liquid chromatography – mass spectrometry. Unsupervised hierarchical cluster analysis of the 171 reliably detected metabolites identified a clear separation based on transplant group (Figure 5(a)), with distinct clusters of metabolites being enriched or depleted in either group. 10 metabolites (5.8%) showed significantly different abundance levels between the FMT-ABX and FMT-C groups, with the majority (adenine, adenosine, betaine, butanoate, 4-coumarate, dihydroxybenzoate, 4-imidazole acetic acid, propionate, pentanoate) being enriched in the FMT-ABX group (Figure 5(b), FDR < 0.05). In contrast, N-Acetyl-L-Alanine was the only metabolite that was significantly enriched in the FMT-C group. Consequently, these findings indicate that a history of antibiotic use can result in significant alterations in microbial metabolism compared with healthy controls.

Among the differentially abundant metabolites, we identified the short-chain fatty acids (SCFAs) propionate, butyrate, and pentanoate. As SCFAs have been associated with gut health and mucosal barrier function,^5,56,57^ we further quantified the SCFA levels in the different groups by targeted SCFAprofiling. Confirming that global profiling (Figure 5(a)), propionic, butyric, and valeric acids were found to be significantly higher in mice who received the FMT from human donors with a history of repeated antibiotic use (*p* < 0.05, Figure 5(c)), which was unexpected with regard to the general assumption that higher levels of SCFAs correlate with improved mucosal barrier function. To thus explore this surprising observation further, we tested for potential correlations between the SCFAs and the most abundant microbial genera. After adjusting for multiple comparisons, we found that *Akkermansia, Butyricimonas*, and *GGB3583* each significantly correlated with propionate (*p_(adj)_* = 0.007, *r_s_* = 0.7853; *p_(adj)_* = 0.008, *r* = −0.7529; *p_(adj)_ *= 0.007, *r_s_ *= 0.8147), butyrate (*p_(adj)_* = 0.013, *r_s_* = 0.7206; *p_(adj)_* = 0.020, *r* = −0.6777; *p_(adj)_* = 0.023, *r_s_* = 0.6765), and valerate (*p_(adj)_* = 0.013, *r_s_* = 0.7206; *p_(adj)_* = 0.007, r = −0.7809; *p_(adj)_* = 0.009, *r_s_* = 0.7529), but not acetate levels, respectively (Figure 5(d)). However, due to the group-dependent differences in CLR abundances for GGB3583, we considered the correlations of this genus as a methodological artifact. Correlations for the remaining most abundant genera were not significant (**Supplementary Figure S5**).

The abundance of *Akkermansia* correlated positively with propionate, butyrate, and valerate production, particularly in the FMT-ABX group where these SCFA concentrations were higher. Interestingly, metagenomic analyses confirmed that the *Akkermansia* genus in our dataset consisted of *Akkermansia muciniphila*, a well-known mucin degrader,^58^ thereby corroborating previous findings that *A. muciniphila* mucolytic activity is associated with the production of SCFAs.^58,59^ However, when testing for correlations within the two individual groups (Figure 5(d), dashed lines) we detected significant positive correlations between propionate (*p* = 0.03, *r* = 0.7561) and butyrate (*p* = 0.0495, *r* = 0.7078) only in the FMT-ABX group, while no significant correlations were observed for the control FMT-C group (propionate *p* = 0.793, r_s_=-0.119; butyrate: *p* = 0.6191, r_s_=-0.2143). Intrigued by this observation, we performed *de novo* metagenomic assembly and identified two distantly related *A. muciniphila* strains, indicated by an average nucleotide identity (ANI) index > 99%, which were distinct between the mice receiving the FMT from donors with a history of antibiotic use and their respective controls (Figure 5(e)). While further characterization of these two strains is required, it is thus possible that repeated antibiotic usage selects for a gut microbial community that is detrimental to mucus function.

## Discussion

Maintaining a safe distance between the gut microbiota and the epithelial barrier is a crucial characteristic of intestinal health. Recently, impaired mucus barrier function has been connected to different diseases, including ulcerative colitis^10,15^ and metabolic disease^14^ in humans and has been associated with cancer development in mice.^60^ Moreover, antibiotic use has been shown to have long-term effects on microbiome composition^18^ and has also been characterized as a risk factor for several diseases such as type-2 diabetes, IBD, and celiac disease.^61–64^ However, while such diseases have been associated with both antibiotic usage and mucus dysfunction, the direct link between the two has so far not been studied. Here, we show that human-derived microbiota from donors with a history of repeated antibiotic use, but no diagnosed disease, can trigger mucus barrier dysfunction in mice, including increased mucus penetrability and reduced mucus growth rate.

Previous studies have highlighted the importance of microbial colonization for normal mucus barrier function^6^ and have implicated certain microbiota and their metabolites in determining Muc2 glycosylation, with conventionally raised mice generally having longer Muc2 glycans than germ-free mice.^65^ Furthermore, microbial composition has also been implicated in mucus layer structure, as demonstrated in studies where the same mouse strain was colonized with different microbiota, resulting in differing mucus function phenotypes.^6,7,43^ Importantly, germ-free mice have an intrinsically defective mucus layer and are thus not optimal for studying mucus function. In our experiments, we thus used mice pre-treated with antibiotics to deplete the inherent mice microbiota. However, while previous studies have not observed any negative impact of acute antibiotic treatment,^6^ a recent study describes a microbiota-independent effect on mucus function by vancomycin treatment.^66^ We can therefore not fully rule out that the mucus layer in our transplanted mice was altered by the prior antibiotic treatment. Still, as both mouse groups were treated similarly, and we still observe significant differences in mucus function between the two groups, it is highly probable that our observed mucus effect is due to the different donor microbiota rather than to a potential effect of previous mouse treatment.

Microbial modulation of mucus function has been shown to follow distinct timelines. A change from a high-fiber diet to a low-fiber Western-style diet leads to reduced mucus growth rate and increased penetrability within 3–7 d after the diet switch.^9^ Likewise, it has been shown that microbial enzymatic mucus degradation activity increases within 1–4 d after a switch to a low-fiber diet,^8^ and our microbial colonization of 10 d after FMT thus allows sufficient time for the development of a mucus phenotype. In contrast, it takes approximately 6 to 8 weeks after microbial colonization of germ-free mice until their penetrable mucus layer becomes impenetrable and reaches the thickness of conventionally raised mice.^6^ This indicates that once the mucus layer is damaged, the presence of beneficial bacteria alone does not immediately affect mucus penetrability, but that a more complex and slower regulatory process, which may include the differential induction of specific glycosyl transferases, is required. Likewise, removing luminal bacteria does not lead to immediate recovery of mucus function, allowing us to capture the mucus function *ex vivo* even after removing the colonic tissue from the animal.

Mucus growth, in contrast, seems to be a faster and more direct effect of the microbiota, and we recently showed that the SCFAs acetate and propionate stimulate mucus growth *ex vivo* through activation of free fatty acid receptor 2 (FFAR2), also termed G-protein coupled receptor 43 (GPR43), within less than 45 min under low dietary fiber feeding conditions.^5^ As mucus growth is thus dependent on the local microbial metabolite profile that is expected to be maintained within the mucus layer after removal from the animal, the mucus growth measured *ex vivo* is expected to reflect the conditions that were present in the mouse gut *in vivo.*

While our analyses focused on mucus function in the distal colon, a recent study highlighted the importance of mucus derived from the proximal colon.^2^ In the mouse gut, mucus produced in the proximal colon encapsulates the fecal pellet and thereby provides a spatial containment of the gut microbiota. This encapsulation is maintained even in the distal colon, thereby adding an “outer” layer of mucus on top of the adherent mucus produced in the distal colon. Thus, while we observed a strong effect of the FMT-ABX gut microbiota on the mucus in the distal colon, it cannot be ruled out that the microbial community causes additional effects also on the mucus of the proximal colon.

To counteract a dysfunctional mucus barrier, the host can activate compensatory mechanisms to reestablish protection against the encroaching antibiotics-induced microbial community. Accordingly, we detected increased mRNA expression of the genes encoding for Muc2 and the antimicrobial peptide Reg3γ (the mouse homologue for human REG3A) in the colon of mice transplanted with the microbiota derived from repeated antibiotic users. The expression of both *Muc2* and *Reg3γ* has been previously shown to be induced by the presence of gut bacteria,^48,67^ and *Reg3γ* has also been shown to be highly expressed in the mouse colon during acute colitis,^68^ suggesting that the ABX-shaped microbiota may induce a similar host response to that observed in the inflamed gut. Importantly, measuring the expression of Muc2 is not a reliable indicator of mucus function, which is rather controlled on a post-translational level and may involve distinct glycosylation and secretion pathways. As such, it was recently shown that colonic mucus secretion is regulated through autophagy and ER stress,^69^ which is not captured by measuring Muc2 gene expression.

Analysis of the microbiota composition in our transplanted mice revealed that several species present in the FMT-ABX group were absent from the microbial community of the FMT-C group, suggesting that antibiotics might be selected for a microbial community that is detrimental to mucus function. Supporting this hypothesis, several species that have previously been shown to degrade mucus differed between the two transplantation groups. Namely, *A. muciniphila*,^58^ *B. fragilis, B. salyersiae, B. producta*, *and P. excrementihominis*,^51,52^ which were more prevalent in the FMT-ABX group when compared to the control group. In contrast, in the FMT-C mice, only *B. thetaiotaomicron*^53^ and *C. cocleatum*^54^ have been previously associated with mucus consumption and, though generally being low in abundance, have higher abundance when compared to the FMT-ABX group.

While moderate mucus consumption is part of a homeostatic microbiota–host interaction, an overly active mucus-foraging microbial community can disturb the balance between mucus secretion and mucus degradation, consequently leading to increased penetrability and barrier breakdown. As such, some bacterial species, including *A. muciniphila*, have been characterized as mucin specialists,^8^ feeding exclusively on mucin O-glycans as a nutrient source, while others have been described as mucin generalists, which are capable of nutrient-dependent flexible foraging. One example of the latter is *B. thetaiotaomicron*, which has a preference for dietary polysaccharides but can switch to feed on mucin O-glycans when dietary nutritional sources are unavailable.^70^ Additionally, *B. thetaiotaomicron* has been shown to produce SCFAs including acetate and propionate,^71^ and we have recently been able to link these SCFAs to improved mucus growth rates under Western-style diet consumption.^5^ Furthermore, *B. thetaiotaomicron* is able to stimulate mucus fucosylation, with induction of fucosylation only occurring if the bacterium is also capable of fucose foraging.^53^ Interestingly, the mucus-promoting abilities of *B. thetaiotaomicron* can be attenuated by the presence of *Faecalibacterium prausnitzii*, demonstrating a homeostatic cross-feeding relationship between bacterial species in the gut in relation to mucus function.^71^

*A. muciniphila*, which is more abundant in the mouse group that received microbiota from donors with a history of antibiotic use, is a well-known mucus degrader, and its excessive colonization has been shown to break the dynamic balance between mucin production and consumption, thereby affecting intestinal barrier function.^72^ Additionally, previous studies have linked *A. muciniphila*-mediated mucus disruption to an exacerbation of food allergy^73^ and *Salmonella typhimurium* infection.^74^ In accordance with our findings, the latter study also detected increased Muc2 expression, but lower numbers of mucin-filled goblet cells in the presence of *A. muciniphila*, confirming that Muc2 expression might not be an optimal indicator of mucin production.

*A. muciniphila*, as well as *B. fragilis*, have been shown to repopulate the colon after antibiotic supplementation.^75,76^ In line with this, we detected *A. muciniphila* in the FMT-C mice in modest abundance, while it reached higher abundance in the FMT-ABX group. Interestingly, using *de novo* metagenomic assembly, we could detect two different previously undescribed *A. muciniphila* strains that were distinct between the mice receiving FMT from donors with a history of antibiotic usage and respective controls. It is thus possible that strain-specific characteristics led to different establishments within the microbial communities, thereby leading to the distinct physiological phenotypes. Besides, a recent study identified that *A. muciniphila* can have discrete functional responses to mucosal barrier function under different dietary conditions: under fiber deprivation, *A. muciniphila* exacerbated susceptibility to the mouse pathogen *Citrobacter rodentium*, whereas it reduced the pathogen load under a fiber-sufficient diet.^77^ This context-dependent behavior might thus be one reason for the controversial role *A. muciniphila* is currently playing in the literature,^78^ as it has, on the one hand, been successfully introduced in a proof-of-concept study as a next-generation probiotic^79^ but, on the other hand, is discussed as a detrimental mucus-degrading species.

Besides differences in the microbial communities, we detected group-specific metabolomics profiles, characterized by significant differences in the abundance of most of the SCFAs. Unexpectedly, propionate, butyrate, and valerate were more abundant in the intestinal content of the FMT-ABX mice, which had a dysfunctional mucus layer. Yet, this may be explained by the fact that most studies that have previously investigated mucus function focused on dietary interventions, often with varying dietary fiber content, which directly affects microbial fermentation and SCFA production^5,8,9^^80,81^ In contrast, when studying mucus function in mouse models of diet-independent obesity, where both groups were fed on a chow diet, no differences in SCFA levels were detected between mouse groups, despite their strong difference in mucus function.^12^ Combined with our present study, these results suggest that SCFAs can potently modulate mucus function under specific environmental conditions, including defined diet-shaped microbial communities, but that high SCFA levels alone are not sufficient to maintain healthy mucus function. As such, *A. muciniphila* has been shown to produce propionic acid as well as stimulate butyric acid production in syntrophic interactions with *Anaerostipes caccae*.^82^ Interestingly, in our experiment *A. caccae* was only detected in the mice who received FMT from donors with a history of antibiotic use. It is thus possible that the co-occurrence of the two species in an antibiotic-disturbed community leads to a microbial composition, in which the SCFA-mediated mucus production^83^ is no longer sufficient to compensate for the excessive mucin degradation by *A. muciniphila*. Alternatively, defects in the intestinal mucosal barrier may impede SCFA-mediated mucus production or SCFA uptake by the intestinal epithelium.

Antibiotic usage early in life increases the risk of developing obesity and central adiposity in humans and mice.^84,85^ Interestingly, we observed significantly higher abdominal fat weight in FMT-ABX mice already 10 d after the microbial transplant. At this time point, however, no difference in body weight was observed. Mucus dysfunction has previously been linked to obesity-associated microbiota in mice, independent of dietary composition,^12^ similar to our current study. Moreover, mice deficient in the antibacterial mucus protein zymogen granule protein 16 (ZG16) displayed enlarged abdominal fat pads, and these enlarged fat pads were not observed when the ZG16^−/−^ mice were depleted of their microbiota.^49^ Consequently, a defective mucus barrier may allow the translocation of commensal intestinal bacteria across the epithelial barrier, which may eventually lead to metabolic impairments.^86^ Such a scenario would thus support the existence of the proposed “gut to adipose tissue axis” and potentially a tissue microbiota in obesity.^87^ Of note, to match the age of the human donors to the recipient mice we used adult mice in this study. Repeating a similar experiment with young mice in the future could provide additional information on whether antibiotic usage early in life may lead to an even stronger mucus phenotype.

A potential limitation of our study is the pooling of four human stool samples for each group for the microbiota transplantations, which creates artificial communities. However, our main aim with this study was to prove the concept that a history of antibiotic usage can shape the microbiota to affect mucus quality. We thus considered this reductionist approach that limited individual variability to improve reproducibility. In humans, pooling donor stool samples for FMT has been shown to induce clinical remission and endoscopic improvement in patients with active ulcerative colitis, in at least two randomized controlled trials.^88,89^ Remarkably, ulcerative colitis is also characterized by a dysfunctional mucus layer^10,15^ and early-onset IBD has been linked to antibiotic usage,^25^ so we are confident that, despite its limitation, our approach is appropriate in this context. However, replicating our findings by using individual stool samples for microbiota transplantations would be required to generalize our findings from this proof-of-concept study. Likewise, due to limited sample availability from this deeply phenotyped cohort, the human-to-mouse microbiota transplantation could not be performed in an independent mouse cohort, which would support further generalization of our findings.

In conclusion, we here identified that a previous – but not recent – history of antibiotic use in humans shaped a microbial community that was insufficient to maintain proper mucus function in mice. Since both intestinal mucosal barrier dysfunction and antibiotic use are increasingly linked to modern lifestyle diseases, including inflammatory bowel disease and metabolic disease, our findings now provide a possible link between these observations. However, despite the usage of human-derived microbiota, further studies are required to verify that repeated antibiotic use causes similar microbiota-mediated mucus defects in humans.

## Supplementary Material

Supplementary Figure 3.tiff

Supplementary Figure 1.tiff

SupplementaryTables.xlsx

Supplementary Figure 4.tiff

Supplementary Figure 5.tiff

Supplementary Figure 2.tiff

## Data Availability

Human stool samples used in this study have been collected from a previously published study,^18^ and the corresponding shotgun metagenomic data have been deposited previously in the European Genome-Phenome Archive database (https://www.ebi.ac.uk/ega/.) under accession code EGAS00001008448. Due to the sensitive nature of the human phenotype data, access is restricted and must be requested through the Estonian biobank. Such access must follow informed consent regulations set out by the Estonian Committee on Bioethics and Human Research (https://genomics.ut.ee/en/content/estonian-biobank.). Preliminary requests for phenotype and raw metagenome data access must be sent to releases@ut.ee. The shotgun metagenomic sequencing data from mice and human pooled samples (human reads removed) are deposited in the European Nucleotide Archive with accession number PRJEB72415 (https://www.ebi.ac.uk/ena/browser/view/PRJEB72415.).
