# Supplementary figures and images for: A history of repeated antibiotic usage leads to microbiota-dependent mucus defects
Source: Gut Microbes. 2024 Jul 21;16(1):2377570. doi: 10.1080/19490976.2024.2377570 (PMC11529412; doi:10.1080/19490976.2024.2377570)

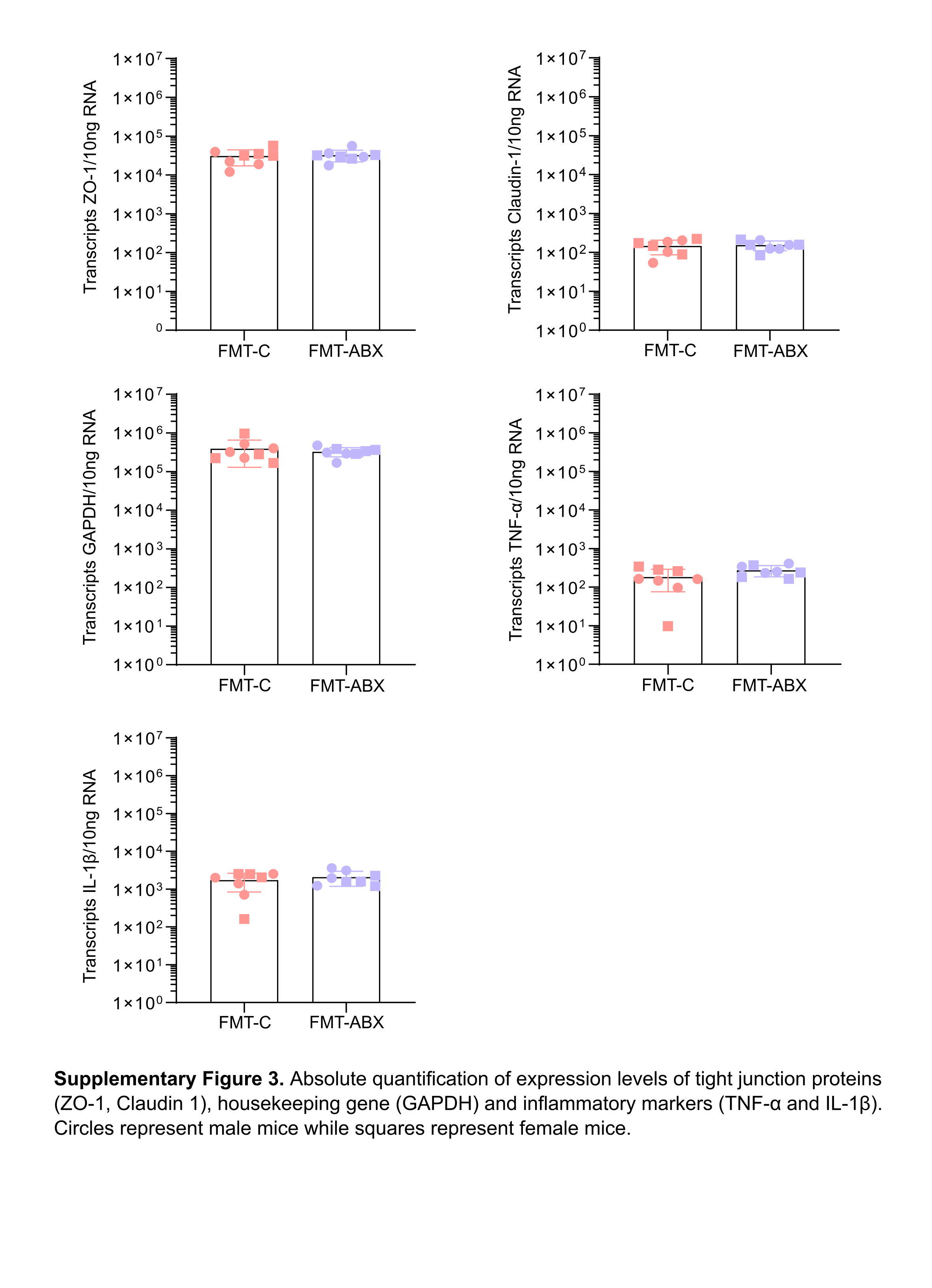

Supplement: Supplementary Figure 3.tiff [file KGMI_A_2377570_SM4735.tiff]

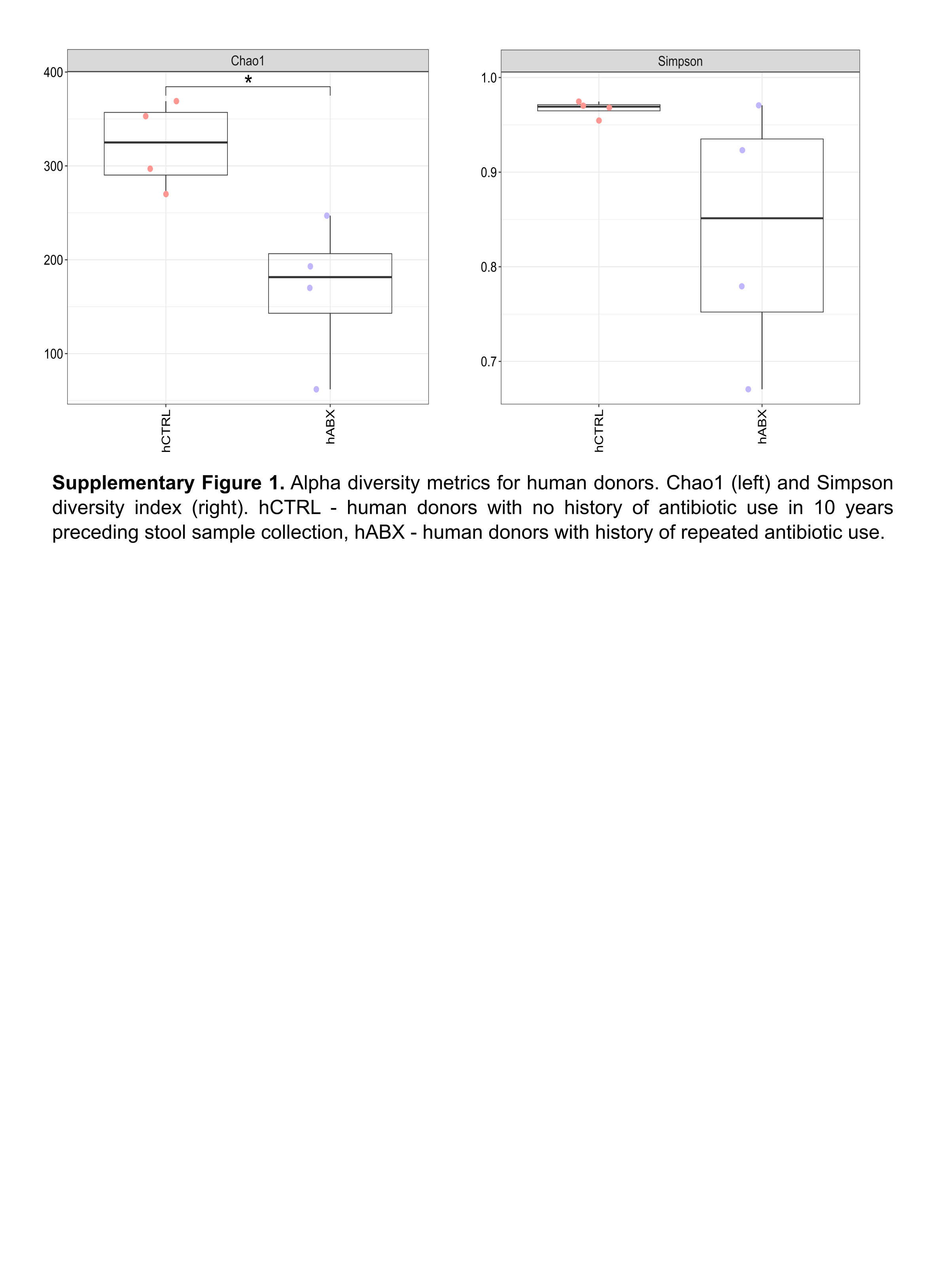

Supplement: Supplementary Figure 1.tiff [file KGMI_A_2377570_SM4734.tiff]

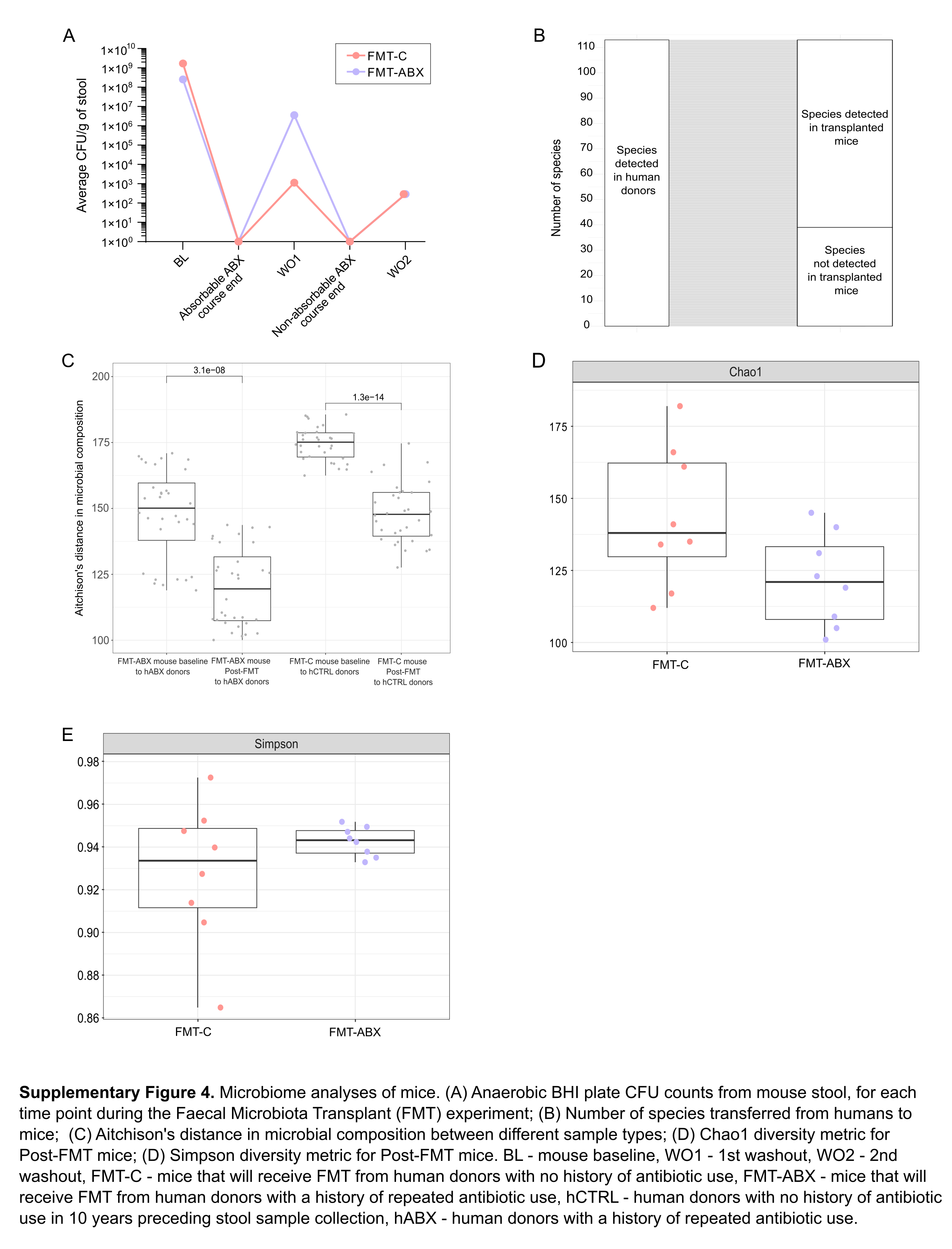

Supplement: Supplementary Figure 4.tiff [file KGMI_A_2377570_SM4732.tiff]

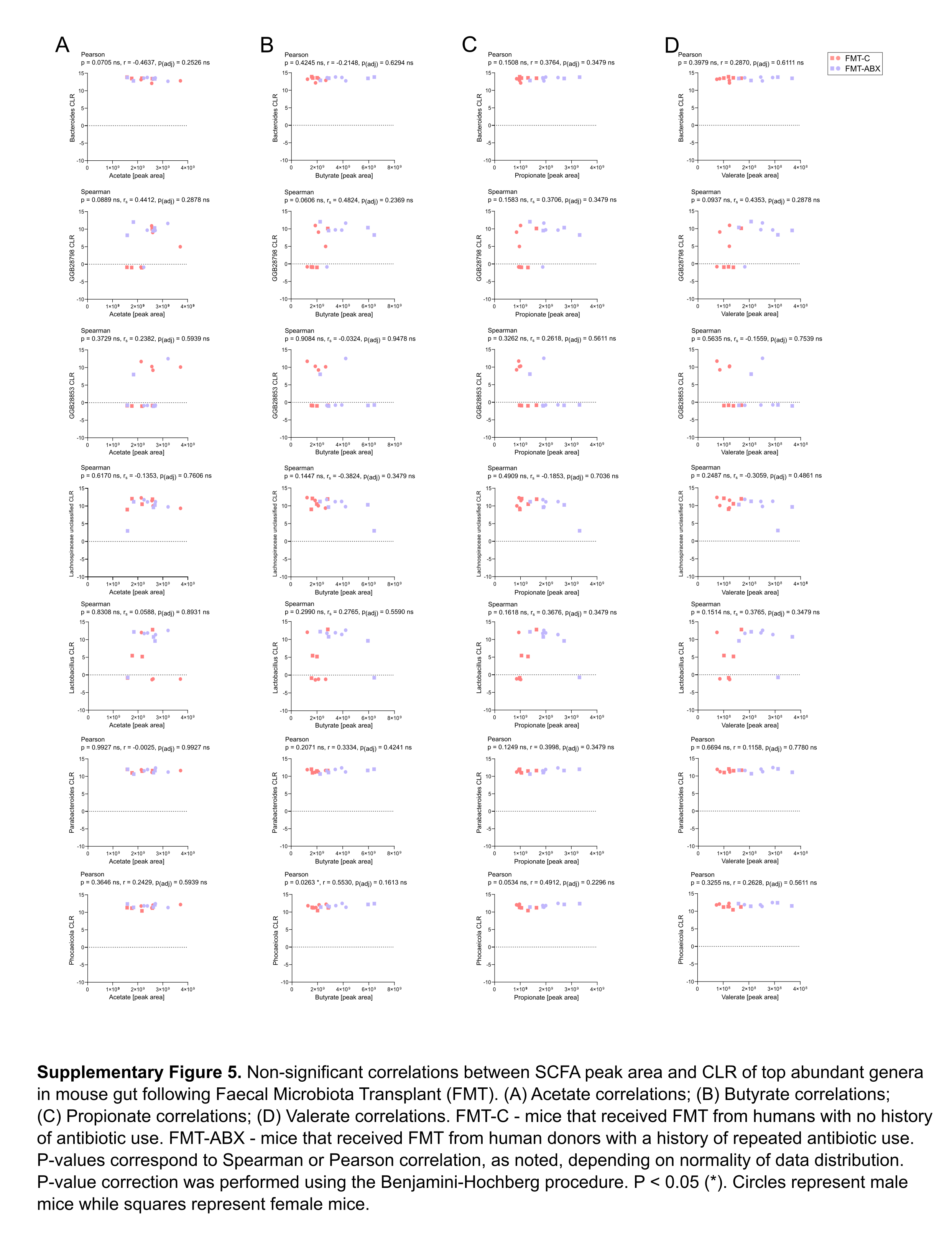

Supplement: Supplementary Figure 5.tiff [file KGMI_A_2377570_SM4731.tiff]

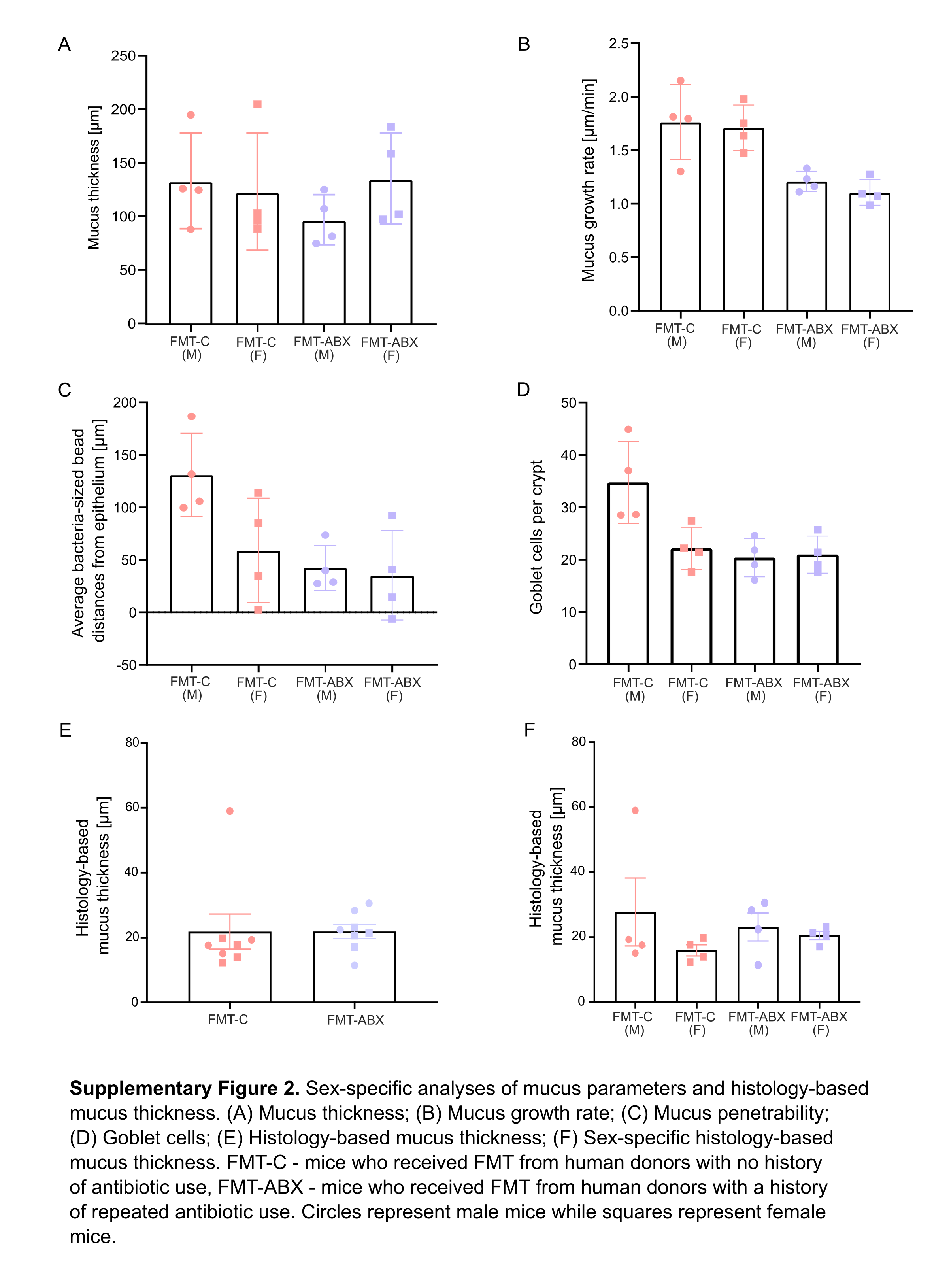

Supplement: Supplementary Figure 2.tiff [file KGMI_A_2377570_SM4730.tiff]
